# Freiburg Neuropathology Case Conference: Blurred Vision and Headaches in a 15-year-old Boy

**DOI:** 10.1007/s00062-020-00973-4

**Published:** 2020-11-23

**Authors:** S. Doostkam, U. Würtemberger, V. Coenen, H. Urbach, M. Prinz, C. A. Taschner

**Affiliations:** 1 Department of Neuropathology, Medical Centre—University of Freiburg, Faculty of Medicine, University of Freiburg, Freiburg, Germany; 2 Department of Neuroradiology, Medical Centre—University of Freiburg, Faculty of Medicine, University of Freiburg, Breisacherstraße 64, 79106 Freiburg, Germany; 3 Department of Stereotactic and Functional Neurosurgery, Medical Centre—University of Freiburg, Faculty of Medicine, University of Freiburg, Freiburg, Germany

**Keywords:** Pineoblastoma, Pineocytoma, Germinoma, Teratoma, Tectal glioma

## Case Report

**Fig. 1:**
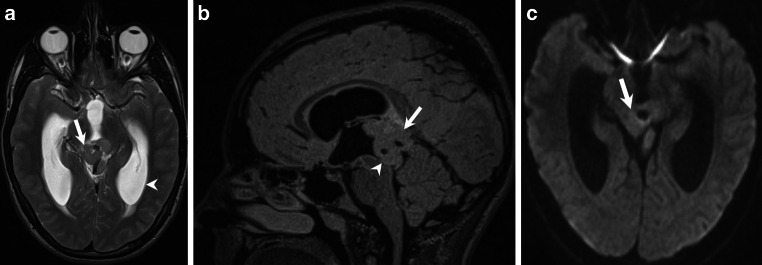
Axial T2-weighted images (**a**) show an isointense lesion (*arrow*) located in the pineal region which lead to subsequent triventricular dilatation (*arrowhead*). On sagittal FLAIR (fluid-attenuated inversion recovery) images (**b**) the lesion (*arrow*) compresses the aqueduct (*arrowhead*). On axial diffusion-weighted images (**c**) the lesion (*arrow*) does not show any signs of restricted diffusion

**Fig. 2:**
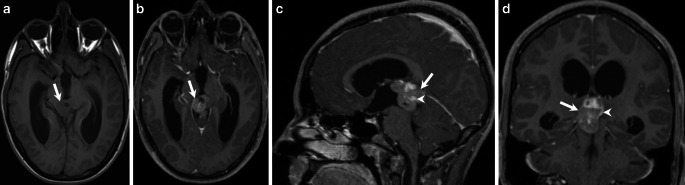
On nonenhanced axial T1-weighted images (**a**) the lesion (*arrow*) is isointense when compared to the brain parenchyma and does not show any signs of infiltration of the surrounding tissue. After administration of gadolinium the lesion (**b**–**d**, *arrow*) shows marked enhancement on axial (**b**), sagittal (**c**), and coronal (**d**) T1-weighted images. Note the deep cerebral veins entrapped within the tumor matrix (**c**, *arrowhead*) and the delicate capsule surrounding the lesion (**d**, *arrowhead*)

**Fig. 3:**
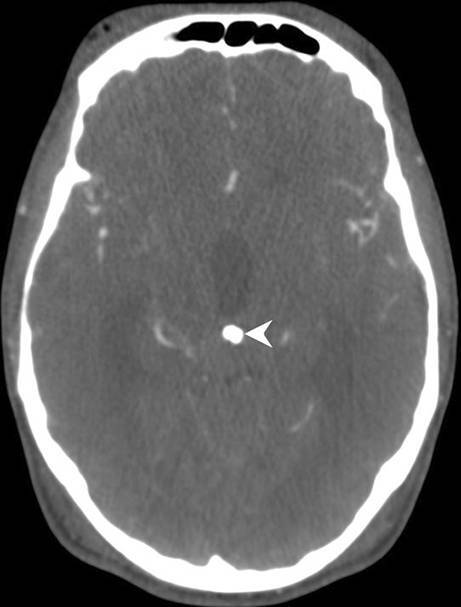
On axial postcontrast computed tomography (CT) images the lesion shows a firm, centrally located calcification (*arrowhead*)

**Fig. 4:**
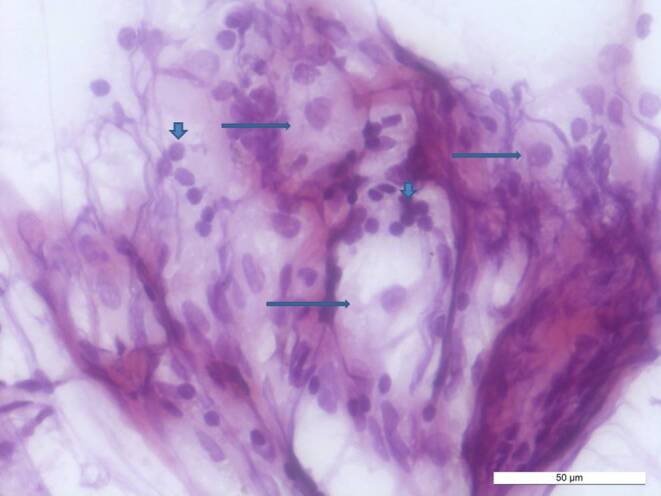
Intraoperative smear preparation, hematoxylin eosin (HE) recolored, shows large tumor cells with broad, weakly eosinophilic cytoplasm (*horizontal arrows*) and small, mature reactive lymphocytes (*vertical arrows*). Scale bar 50 µm

This 15-year-old boy was diagnosed with papilledema and papillary venous bleeding diagnosed as a result of a work-up after 6–9 months of persistent headaches, which typically occurred twice a week. The character was pounding and at times reached 6–7 points on a 10-point intensity scale. Besides blurred vision and headaches the neurological examination was non-focal, especially no problems passing urine or mental decline were detected. A pineal region tumor was found on brain magnetic resonance imaging (MRI, Figs. 1, 2, 3 and 4). Blood samples were negative for alpha-fetoprotein (AFP) and beta-human chorionic gonadotropin (beta- HCG).

After interdisciplinary discussion in the tumor board an endoscopic third ventriculostomy was performed together with a stereotactic biopsy of the contrast enhancing mass of the posterior third ventricle.

The postoperative course was uneventful and after surgery the patient recovered to the normal baseline with no headaches and improved vision.

## Imaging

On axial T2-weighted images (Fig. 1a) an isointense lesion (arrow) located in the pineal region was found which led to subsequent triventricular dilatation (arrowhead). On sagittal FLAIR images (Fig. 1b) the lesion (arrow) compresses the aqueduct (arrowhead). The lesion (Fig. 1c, arrow) does not show any signs of restricted diffusion on diffusion-weighted images (DWI). On nonenhanced axial T1 weighted images (Fig. 2a) the lesion (arrow) is isointense when compared to the brain parenchyma and does not show any signs of infiltration of the surrounding tissue. After administration of gadolinium the lesion shows marked enhancement (Fig. 2b–d, arrow). Note the deep cerebral veins entrapped within the tumor matrix (Fig. 2c, arrowhead) and the delicate capsule surrounding the lesion (arrowhead, Fig. 2d). On postcontrast computed tomography (CT) images the lesion shows a firm, centrally located calcification (Fig. 3, arrowhead).

## Differential Diagnosis

### Pineoblastoma

Pineoblastomas are highly malignant embryonal World Health Organization (WHO) grade IV tumors of the pineal gland, which clinically manifest with symptoms of elevated intracranial pressure due to aqueduct obstruction and subsequent obstructive hydrocephalus, such as nausea and vomiting. When compression of the superior tectal plate is present, it may also lead to Parinaudʼs syndrome—a combination of supranuclear vertical gaze disturbance [1]. These tumors most frequently occur in young children with a slight female predominance. The typical imaging features consist of a poorly defined large mass in the pineal region, very often measuring >3 cm and infiltrating bordering brain structures [2, 3]. On nonenhanced CT images, pineoblastomas present as large masses with peripherally scattered calcifications and relative hyperdense solid components making it often difficult to distinguish from pineocytoma [4]. Magnetic resonance imaging is more appropriate to demonstrate infiltration of adjacent brain structures and shows heterogeneous hypointensity and isointensity in T1-weighted and T2-weighted sequences of the solid tumor parts, which exhibit diffusion restriction and have significantly lower ADC (apparent diffusion coefficient) values compared to germinomas. Very often necrosis and cysts can be encountered, as well as a mild perifocal edema on T2/FLAIR. Heterogeneous contrast enhancement is seen and calcifications manifest as blooming on susceptibility weighted images (SWI) [3, 5]. Due to frequent tumor dissemination into the cerebrospinal fluid (CSF), imaging of the entire neuroaxis must be performed. The “exploded” peripheral calcifications typically seen in pineoblastoma are not present on CT images in our case, making this diagnosis less likely.

### Pineocytoma

Pineocytomas are relatively benign WHO grade I tumors, which can be encountered at any age but tend to occur more commonly in adults. Comparable to other pineal region masses, clinical presentation is mainly caused by tectal compression including the superior colliculi as well as aqueduct obstruction with hydrocephalus. Tumor size is an important feature for discrimination from pineoblastoma, as the former usually is smaller (<3 cm). Otherwise, CT imaging is very helpful as it displays the peripheral “exploded” calcifications corresponding to the pre-existing pineal calcifications [1] and both CT and MRI can show avid, heterogeneous solid or peripheral enhancement in a well-circumscribed, FLAIR/T2-hyperintense pineal mass [1, 3, 5]. Cystic components might be present and could mimic a pineal cyst. The primary treatment consists of surgical removal, which leads to high survival rates when completely resected. Tumor size and the lack of peripherally dispersed calcification make this diagnosis less likely in the present case.

### Germinoma

Pineal germinomas or intracranial germ cell tumor (IGCT) are the most common tumors of the pineal region and in most cases encountered in young patients <20 years old, with a strong male predominance, whereas suprasellar location is more frequently found in women [1, 6, 7]. Pineal germinomas are WHO grade II tumors considered to descend from persistent dysplastic germ cells and demonstrate increased beta-HCG and placental alkaline phosphatase (PLAP) in the laboratory analyses. When located in the pineal region, clinical symptoms resemble other pineal tumors, which include visual disturbances and signs of elevated intracranial pressure. Nonenhanced CT can play a pivotal role showing characteristic central calcification (engulfment), which can correspond to both surrounded pineal calcification and tumor calcification. MRI shows cortex isointense T1 and T2 signal intensity with vivid but generally homogeneous contrast enhancement and very often infiltration of adjacent brain parenchyma. DWI can mirror high tumor cellularity with diffusion restriction, but with higher ADC values than pineoblastomas. Multiple cysts and less commonly hemorrhage can also be found [6, 7]. Germinomas can also be located in the suprasellar region or less commonly in the basal ganglia and thalami. Hallmark signs in this location are the absence of the bright spot of the posterior pituitary and presence of a prominent pituitary stalk. In these cases, clinical symptoms can include early diabetes insipidus and disturbance of the hypothalamic-pituitary function. Furthermore, CSF seeding is common, requiring imaging of the entire neuroaxis in germinoma patients. The combination of tumor location, apparent central calcification in CT and the contrast enhancement in MRI as well as the patient’s age, sex and clinical symptoms made pineal germinoma the most probable diagnosis in our patient.

### Teratoma

Intracranial teratomas are nongerminomatous germ cell tumors that can be classified as mature, immature or mature with malignant transformation as well as intra-axial or extra-axial and are despite their rare prevalence in children the most common perinatal brain tumors. Whereas intra-axial teratoma can generally be diagnosed in utero by ultrasound, the extra-axial teratomas tend to occur in younger adults or in childhood with male predominance and are frequently located in the pineal region or other midline structures such as the (supra)sellar region or basal ganglia. As teratoma are comprised of elements from different germinal layers, they typically exhibit mixed tissue with very heterogeneous imaging characteristics; however, most of them contain calcifications, fat and soft tissue elements as well as cysts [8, 9]. When present in the pineal region, teratomas exert mass effect on the tectum. On CT, the fat and calcification elements can be easily visualized and MRI sequences should be acquired with and without fat suppression. Calcification leads to signal drop in SWI sequences as well as variable signal intensity in T1-weighted images, whereas the fat tissue components increase the T1 signal. The solid tumor components are diffusion restricted when highly cellular and show contrast enhancement, which facilitates differentiation from lipoma or dermoid cysts [8, 9]. Since we did not find any signs of fatty tissue within the lesion, a teratoma can be discarded.

#### Tectal Glioma

A subgroup of brainstem gliomas, tectal glioma are rare low-grade tumors predominantly manifesting in children and young adults complaining about headaches caused by aqueduct stenosis and should be considered as differential of pineal region masses in rare cases when tectal gliomas are larger sized, invasive and not clearly nascent from the tectal plate [10]. MRI typically demonstrates a nonenhancing, T1-hypointense and T2-hyperintense lesion with “T2 shine through” phenomenon on DWI. Due to their relatively benign course, CSF diversion is very often the only necessary treatment for symptom relief. In the rare cases of progress, contrast enhancement and cystic lesions are encountered [10–12]. In our case the clear enhancement made this diagnosis less likely.

## Histology

**Fig. 5:**
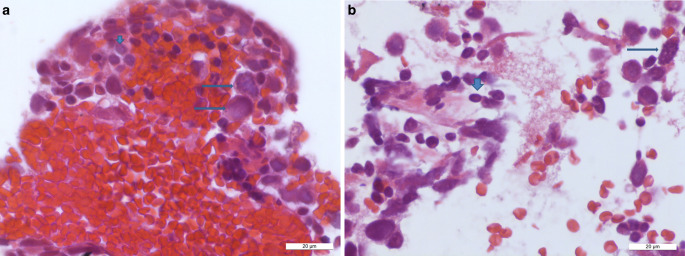
The HE preparations of the formalin-fixed and paraffin-embedded material show predominantly freshly coagulated blood with some large tumor cells (**a**, *horizontal arrows*), in **b** a mitotic figure (*horizontal arrows*) and some small mature lymphocytes (*vertical arrow*). Scale bars 20 µm

In addition to gliotic central nervous tissue, many lymphoid cells as well as some larger cells with a relatively clear and broad cytoplasm were detectable in the intraoperative smear preparations (Fig. 4). Some of the larger cells showed prominent nucleoli. In the formalin-fixed and paraffin-embedded stereotactic samples, almost fresh coagulated blood was present. Within the blood, however, as well as in the intraoperative smear preparations, some larger tumor cells with a broad cytoplasm and prominent nucleoli were recognizable in hematoxylin and eosin staining (HE, Fig. 5a, b). A mitotic figure could also be found here (Fig. 5b). Small fragments of gliotic CNS tissue appeared on the margins.

**Fig. 6:**
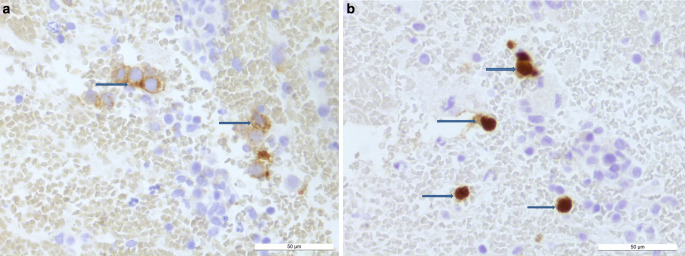
Immunohistochemistry for c‑Kit (**a**) and Oct 3/4 (**b**), the tumor cells show immunoreactivity for c‑Kit and Oct 3/4 (*horizontal arrows*). Scale bars 50 µm. 4

**Fig. 7:**
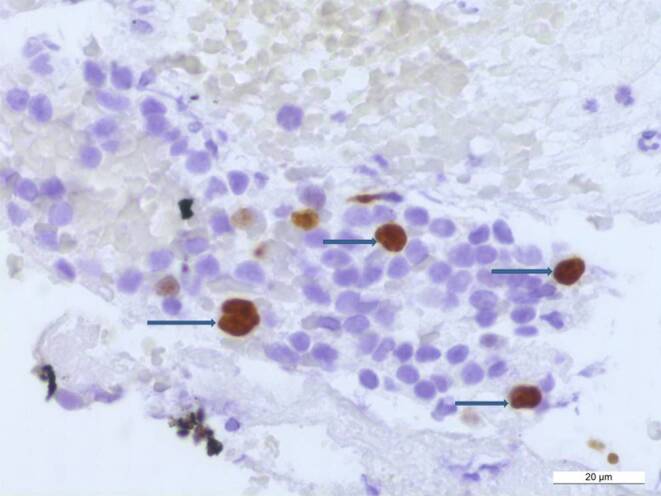
The tumor cells show a significantly increased proliferation rate in the MIB1 immunohistochemistry: Almost all large cell nuclei are positively marked (arrows). Scale bar 20 µm

The tumor cells showed immunoreactivity for Oct3/4 (Fig. 6a) and c‑Kit (Fig. 6b). The immunohistochemical reactions for glial fibrillary acidic protein (GFAP), synaptophysin, neuron-specific enolase (NSE), neurofilament (NF), pancytokeratin (AE1/3), beta-HCG, AFP, PLAP were negative in the tumor cells. Leukocyte common antigen (LCA) labeled some leukocytes. The proliferation rate in the MIB1 (Ki67) immunohistochemistry was clearly high within the tumor cells (Fig. 7). The diagnosis of germinoma was made on the basis of the histopathological and immunohistochemical results. There was no evidence of a pineal tumor.

Stained specimens and blank sections were sent to the brain tumor reference center in Bonn for reference pathological assessment, where our initial diagnosis of germinoma was confirmed.

## Diagnosis

### Germinoma (WHO Grade II)

Intracranial germ cell tumors are rare malignant diseases of the central nervous system and occur primarily in the central and middle structures of the brain, but most frequently in the pineal and suprasellar regions [13, 14]. They show strong regional differences in incidence and are much more common in East Asia than in Europe and the USA [13, 15]. The highest incidence occurs in patients aged 10–14 years, and a clear majority of cases of all histological types involves males [14, 16]. Germ cell tumors generally originate from cells of very early embryonic development, so-called totipotent primordial germ cells. The midline localization of these tumors corresponds to the migration of the original cells in the embryonic development [14, 16].

Germinomas are the most common intracranial germ cell tumors. They make up about two thirds of all intracranial germ cell tumors and are histologically comparable to the dysgerminoma of the ovaries and the seminoma of the testicles [13, 16]. They develop from undifferentiated germinal epithelia and are extremely sensitive to radiotherapy [17, 18]. If a diagnosis cannot be made unequivocally by CSF analysis, histological confirmation by means of a biopsy is indicated. Surgical treatment in the sense of resection therefore plays a subordinate role here and is limited to a histological confirmation of the diagnosis.
